# Urinary tract infection in a cat and cholangiohepatitis in a dog caused by the same strain of CTX-M-14 extended-spectrum beta-lactamase-producing *Escherichia coli* ST162 in a household: case report

**DOI:** 10.1128/asmcr.00069-25

**Published:** 2025-08-26

**Authors:** Tessa E. LeCuyer, Alexandra S. Fox, Amanda A. Carbonello, Diamond McClendon, Timothy Bolton

**Affiliations:** 1 Department of Pathology, Microbiology & Immunology, School of Veterinary Medicine, University of California, Davis 70733, Davis, California, USA; 2 Virginia Tech Animal Laboratory Services, Virginia-Maryland College of Veterinary Medicine, Virginia Tech 70732, Blacksburg, Virginia, USA; 3 Department of Veterinary Clinical Sciences, Purdue University College of Veterinary Medicine 311308 , West Lafayette, Indiana, USA; Vanderbilt University Medical Center, Nashville, Tennessee, USA

**Keywords:** transmission, household, ESBL

## Abstract

**Background:**

Extended-spectrum beta-lactamase (ESBL)-producing Enterobacterales can be disseminated within households, including sharing of strains between people and pets, posing a public health risk. This case report demonstrates that household dissemination can also lead to antimicrobial-resistant infections in other pets in the household.

**Case Summary:**

A 14-year-old domestic shorthair cat with a subcutaneous ureteral bypass system was diagnosed with a urinary tract infection caused by *Escherichia coli* ST162 with CTX-M-14 ESBL. The cat was successfully treated. Seven months later, a 16-year-old mixed-breed dog in the same household was diagnosed with cholangiohepatitis caused by *E. coli* ST162 with CTX-M-14. Genomic comparison of the feline urine and canine bile isolates showed that they were highly genetically similar, despite months passing between resolution of the cat’s infection and the beginning of the dog’s clinical signs.

**Conclusion:**

Dissemination of ESBL-producing *E. coli* to other pets, as well as to people, is possible when an animal in the household has an infection. In this case, cohabiting animals both developed clinical infections, highlighting the need for guidance to prevent pet-to-pet spread of antimicrobial-resistant pathogens.

## INTRODUCTION

Extended-spectrum beta-lactamase (ESBL)-producing Enterobacterales (E) are a threat to public health, and nosocomial transmission is documented in both human and veterinary healthcare settings (1–3). However, in people, the rate of ESBL-E transmission within the household exceeds that of transmission between hospitalized patients (4). Even when there is household spread of ESBL-E, introduction of the strain into the household may begin with the hospitalization event of a single person, leading to dissemination of gastrointestinal carriage throughout the household (5). Dogs and cats can also become colonized with ESBL-E during hospitalization, and humans in the household may become gastrointestinal tract carriers with the same ESBL-E strain as their pet once the pet is back in the home (6, 7).

While there is evidence of gastrointestinal ESBL-E sharing between people and their pets, the relationship between gastrointestinal carriage and the subsequent development of clinical infections is poorly understood (8–12). Gastrointestinal tract carriage of ESBL-E often precedes clinical infections at extraintestinal sites, but the number of people and pets who are ESBL-E carriers that eventually go on to develop clinical infections is low (11–13). Because of the public health threat of ESBL-E, most studies investigate human-pet sharing of ESBL-E rather than considering the risks to animal health that these organisms pose when they cause extraintestinal infection. In this case report, we describe household sharing of a strain of ESBL *Escherichia coli* that caused clinical infections in both a cat and then a dog in the same household.

## CASE PRESENTATION

**Fig 1:**
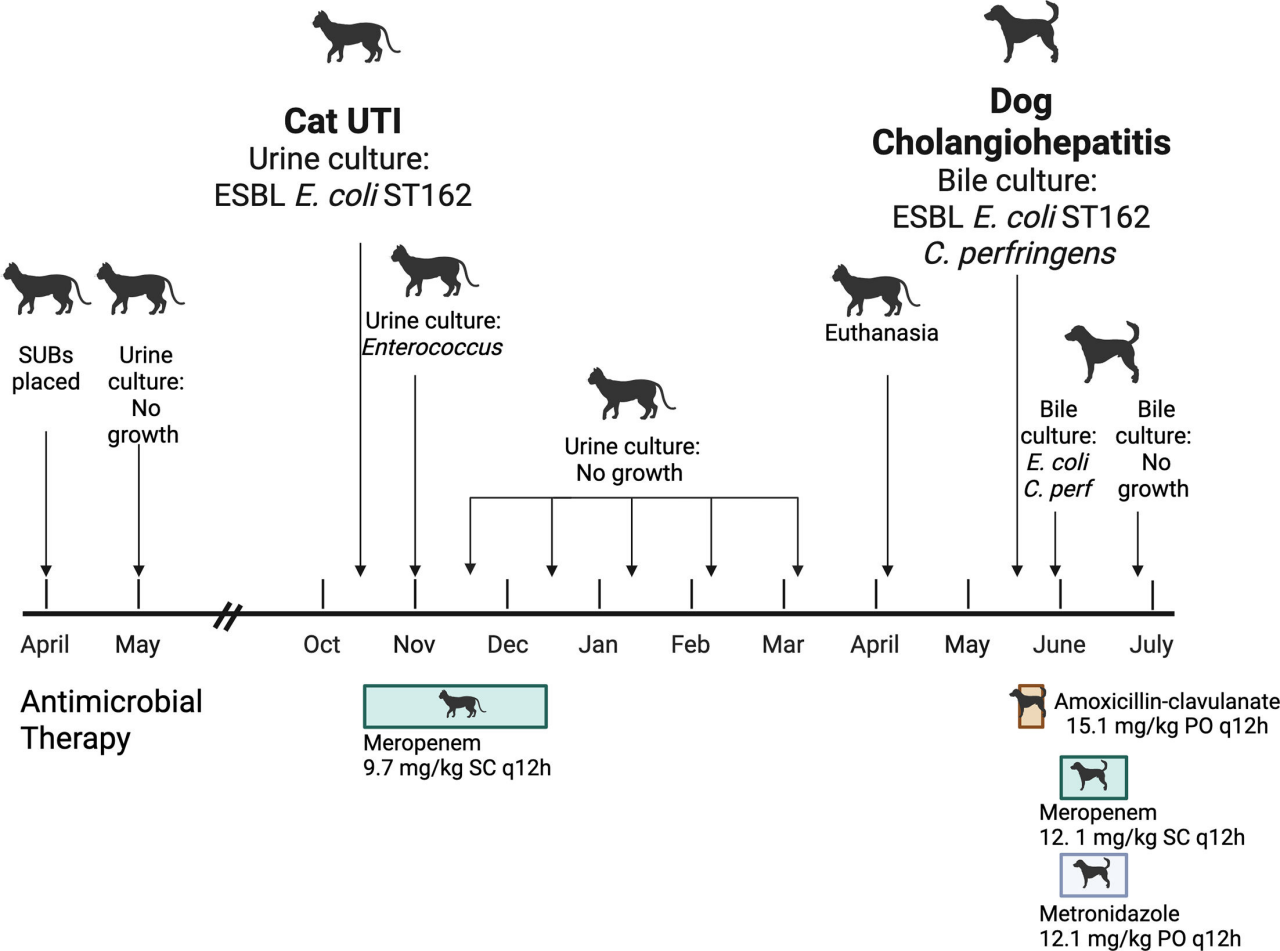
Timeline of events in the diagnosis and treatment of a strain of extended spectrum beta-lactamase-producing (ESBL) *E. coli* ST162 isolated from a cat with UTI and a dog with cholangiohepatitis in the same household. Figure created with BioRender.com/oij8nr8.

A 14-year-old female domestic shorthaired cat presented for a flush of her subcutaneous ureteral bypass (SUB) systems. A few days preceding this visit, the cat became lethargic and hyporexic and produced small amounts of grossly normal urine in the litterbox. The SUB systems were placed in the urinary tract 6 months prior as treatment for bilateral obstructive ureteroliths. Urine collected in a sterile manner from the SUB systems contained large numbers of leukocytes (100–150 per hpf), and an aerobic bacterial culture yielded heavy growth of *E. coli*. The organism was phenotypically resistant to all tested penicillins, cephalosporins, fluoroquinolones, tetracyclines, sulfonamides, and chloramphenicol. Thus, the cat was treated with meropenem (Merrem, Pfizer) 9.7 mg/kg by subcutaneous injection (SC) q12h for 8 weeks. Aerobic bacterial cultures of urine obtained from the SUB systems at 4, 8, 11, 15, and 20 weeks subsequent to the initiation of meropenem yielded no bacterial growth (Fig. 1).

The cat had hyperthyroidism treated with radioactive iodine 2.5 years prior. Following treatment, chronic renal disease (Stage II) was unmasked and stable at the time of the SUB flush. Additionally, the cat had idiopathic hypercalcemia successfully controlled with chia seed supplementation of a commercial prescription renal diet. Finally, the cat had no history of antimicrobial administration in the past 2.5 years, and urine collected in a sterile manner from the SUB systems 5 months prior to the flush visit (1 month following SUB systems placement) demonstrated no bacterial growth. The cat was euthanized 6 months following the diagnosis of the *E. coli* infection due to progressive azotemia that was believed unrelated to the *E. coli* infection because of the series of negative urine cultures shortly preceding this.

A 16-year-old male mixed-breed dog presented for vomiting, inappetence, and fever approximately 7 months after the cat in the same household was diagnosed with an *E. coli* infection of the urinary tract. On physical examination, the dog had faintly icteric sclera. Serum chemistry demonstrated increases in liver enzymes and total bilirubin consistent with hepatobiliary disease. Bile obtained through ultrasound-guided cholecystocentesis contained filamentous and spore-forming short rod-shaped bacteria. Pending the bile culture results, the dog was treated with amoxicillin-clavulanate (Clavamox, Zoetis) 15.1 mg/kg by mouth (PO) q12h.

Aerobic and anaerobic bacterial cultures of the bile yielded heavy growth of both *E. coli* and *Clostridium perfringens*. The *E. coli* was phenotypically resistant to all tested penicillins and cephalosporins, apart from ceftazidime and piperacillin-tazobactam, fluoroquinolones, tetracyclines, sulfonamides, and chloramphenicol. Thus, the dog was switched from amoxicillin-clavulanate to meropenem 12.1 mg/kg SC q12h and metronidazole (Flagyl, Pfizer) 12.1 mg/kg PO q12h for 3 weeks. Aerobic and anaerobic bacterial culture of the bile following completion of dual antibiotic therapy yielded no bacterial growth (Fig. 1).

The dog had two discrete episodes of idiopathic vestibular disease in the 3 months preceding the biliary tract infection from which it fully recovered. Additionally, the dog had stable chronic renal disease (Stage I) for which it was fed a commercial prescription renal diet. Lastly, the dog had no history of antimicrobial administration in the 3 months since evaluation for the vestibular disease episodes. Due to owner relocation, the dog was lost to follow-up.

### Bacterial characterization

Specimens from both patients were submitted to Virginia Tech Animal Laboratory Services (Blacksburg, VA) for diagnostic bacteriology testing. The organisms were identified by matrix-assisted laser desorption ionization time-of-flight mass spectrometry (Biotyper, Bruker, Billerica, MA). Antimicrobial susceptibility testing was performed by broth microdilution using Sensititre COMPGN1F plates and read on a Sensititre Vizion instrument (Trek Diagnostic Systems, Thermo Fisher Scientific, Oakwood Village, OH). Canine and feline Clinical and Laboratory Standards Institute breakpoints for Enterobacterales were used for interpretation when available (14). The bacterial isolates from both patients were banked in tryptic soy broth with 18% glycerol at −80°C. *E. coli* isolates from both patients were recovered from frozen stocks on Columbia blood agar (Remel, Thermo Fisher Scientific, Lenexa, KS), and DNA was isolated using a DNeasy Blood and Tissue Kit (Qiagen, Germantown, MD). DNA libraries were prepared (Illumina DNA Prep and Nextera DNA CD Indexes, Illumina, San Diego, CA) and sequenced on an Illumina iSeq 100 instrument (Illumina, San Diego, CA). Raw reads were uploaded to GalaxyTrakr, and the MicroRunQC workflow was run (15). Both isolates had adequate quality control parameters (coverage >40×, <300 contigs, average read quality Q scores >30, and sequence lengths ~4.8 Mbp). Antimicrobial resistance genes and plasmid types were determined with the staramr workflow and filtered core genome single nucleotide polymorphisms (SNPs) were identified by the CFSAN SNP pipeline using *E. coli* K-12 MG1655 ASM584v2 (RefSeq GCF_000005845.2) as a reference (16, 17). Plasmid sequences were extracted using MOB-Recon (18, 19).

The *E. coli* isolates from both patients were highly similar, despite being isolated 7 months apart. A summary of the isolates is provided in Table 1. The major differences between the genomes of the two organisms were 4 SNPs, located at the IS3 family transposase, the molecular chaperone HtpG, the mechanosensitive channel MscK, and the loss of an IncF plasmid that was present in the feline isolate but not in the canine isolate. Sequences associated with the IncF plasmid solely in the feline isolate contained a 1,248 bp region that was 100% identical to *bla*_CMY-2_ (GenBank WP_000976514.1). The phenotypic susceptibility patterns of the two organisms differed slightly, primarily in ceftazidime and piperacillin-tazobactam susceptibility, presumably due to the loss of *bla*_CMY-2_ in the canine isolate.

**TABLE 1:** Summary of the *E. coli* ST162 isolated from clinical infections of a cat and a dog living in the same household^*a*^

	Cat		Dog	
Site of infection	Urinary tract (including SUB device)		Gallbladder and liver	
Antimicrobial resistance	Phenotypic	Genotypic	Phenotypic	Genotypic
Aminoglycoside	None	*aph (6)-Id, ant(3″)-Ia*	None	*aph (6)-Id, aadA5, aph(3″)-Ib*
Beta-lactam	Resistant (ampicillin, amoxicillin-clavulanate, cefazolin, cefpodoxime, ceftazidime, and piperacillin-tazobactam) susceptible to imipenem	*CTX-M-14, TEM-1B, CMY-2*	Resistant (ampicillin, amoxicillin-clavulanate, cefazolin, and cefpodoxime) susceptible to imipenem, ceftazidime, piperacillin-tazobactam	*CTX-M-14, TEM-1B*
Tetracycline	Resistant	*tet(B*)	Resistant	*tet(B*)
Trimethoprim-sulfamethoxazole	Resistant	*sul2, dfra17*	Resistant	*sul2, dfra17*
Fluoroquinolone	Resistant (enrofloxacin, marbofloxacin, orbifloxacin, and pradofloxacin)	*gyrA* (S83L), *parC* (S80I)	Resistant (enrofloxacin, marbofloxacin, orbifloxacin, and pradofloxacin)	*gyrA* (S83L), *parC* (S80I)
Chloramphenicol	Resistant	*catA1*	Resistant	*catA1*
Disinfectants	Not tested	*sitABCD*	Not tested	*sitABCD*
Plasmids present	IncFIB, IncQ1, IncFII or IncFIA, IncFIC		IncFIB, IncQ1	

^*a*^ Information that is underlined is different between the two isolates. The core genomes of the two isolates varied by four single-nucleotide polymorphisms. Phenotypic antimicrobial resistance was based on broth microdilution and genotypic resistance was based on *in silico* antimicrobial resistance gene detection.

## DISCUSSION

In this case of apparent transmission of ESBL-E that caused clinical infections in a dog and a cat in the same household was only detected because of genomic surveillance of ESBL-E in a veterinary diagnostic laboratory. While cats are less likely to be ESBL-E carriers than dogs, they do occasionally share ESBL-E strains with their owners and with dogs in the household (8–10, 20). Most ESBL-E household sharing does not lead to clinical disease, but a dog with a urinary tract infection (UTI) caused by a strain of *E. coli* ST131 also recovered from the feces of a cat in the same household has been previously reported (21).

Carbapenems, designated as critically important antimicrobials by the World Health Organization, are infrequently prescribed to dogs and cats (22–24). The International Society for Companion Animal Infectious Diseases (ISCAID) offers guidance for determining when carbapenem use is justified in companion animals (25–27). These suggestions were followed for the cases in this series; there was documented phenotypic resistance to many other drug classes that supported the selection of meropenem to treat both patients, and consultation with a clinical microbiologist and a clinical pharmacologist occurred. Additionally, both animals had clinical signs of illness related to their infections, further justifying treatment with an antibiotic known to be effective based on *in vitro* testing. Follow-up negative culture results in both animals indicated that the drug choice was appropriate for a bacteriologic cure.

The clinical signs in the cat described in this study were concerning for a UTI with spread to the kidneys (pyelonephritis). Unfortunately, a diagnosis of pyelonephritis in a cat is only presumptive unless there is a positive bacterial culture from a urine sample obtained from just the renal pelvis via pyelocentesis. In this case, the SUB devices prevented such a procedure from being performed; thus, it was the clinical signs of illness in conjunction with a positive urine culture obtained through the SUB devices that led to a presumptive diagnosis of pyelonephritis. While the 2019 ISCAID guidelines recommend just 2 weeks of antibiotic treatment in cases of pyelonephritis (24), the presence of implants and risk of development of carbapenem resistance if the infection was not fully cleared resulted in election for a longer treatment duration. It should be stated that this treatment duration was based on clinician (T.B.) concerns and not concrete data indicating it was necessary.

A limitation of this report is that the source of the ESBL *E. coli* isolated from the animals in this household is unclear. While raw meat diets are a risk factor for ESBL-E carriage in pets, this is not a probable source as both animals were fed strict species-specific commercial prescription diets (8, 28). Hospitalization is a risk factor for ESBL-E carriage in people and pets, and the cat was hospitalized several times in the years prior to infection (4, 8). It cannot be ruled out that both animals acquired this organism from a common external source, rather than transmitting the organism to one another.

This report highlights the fact that pets with ESBL-E infections are a source of bacteria that can cause clinical infections in other animals in the household, and those clinical infections may occur well after the index case has resolved. As in this case, household dissemination of highly antimicrobial-resistant strains can lead to increased use of critically important antimicrobials in animals. Key transmission events for ESBL-E transfer between animals are currently unknown, and most of the hygiene practices that are recommended to reduce ESBL-E transmission in people, such as handwashing, are not extrapolatable to veterinary patients. However, advice for prevention of zoonotic transfer of resistant pathogens from animals to people, such as prompt waste removal, frequent cleaning of pet supplies and living spaces, and caretaker handwashing after animal handling, could also prevent dissemination between pets (29). Further research is needed to better understand how to limit the spread of ESBL-E between companion animals living in the same household.

## Data Availability

Genomic sequences are available as SRA accession numbers SRR31611264 and SRR31611022.
